# Genomic diversity and distribution of *Bifidobacterium longum* subsp. *longum* across the human lifespan

**DOI:** 10.1038/s41598-017-18391-x

**Published:** 2018-01-08

**Authors:** Toshitaka Odamaki, Francesca Bottacini, Kumiko Kato, Eri Mitsuyama, Keisuke Yoshida, Ayako Horigome, Jin-zhong Xiao, Douwe van Sinderen

**Affiliations:** 1 Next Generation Science Institute, Morinaga Milk Industry Co., Ltd, Zama, Kanagawa Japan; 2 APC Microbiome Institute and School of Microbiology, National University of Ireland, Western Road, Cork, Ireland

## Abstract

*Bifidobacterium longum* subsp. *longum* represents one of the most prevalent bifidobacterial species in the infant, adult and elderly (human) gut. In the current study, we performed a comparative genome analysis involving 145 *B. longum* representatives, including 113 *B. longum* subsp. *longum* strains obtained from healthy Japanese subjects aged between 0 and 98 years. Although MCL clustering did not reveal any correlation between isolated strains and subject age, certain characteristics appear to be more prevalent among strains corresponding to specific host ages, such as genes involved in carbohydrate metabolism and environmental response. Remarkably, a substantial number of strains appeared to have been transmitted across family members, a phenomenon that was shown not to be confined to mother-infant pairs. This suggests that the ubiquitous distribution of *B. longum* subsp. *longum* across the human lifespan is at least partly due to extensive transmission between relatives. Our findings form a foundation for future research aimed at unraveling the mechanisms that allow *B. longum* strains to successfully transfer between human hosts, where they then colonize and persist in the gut environment throughout the host’s lifespan.

## Introduction

Bifidobacteria are natural inhabitants of the human gastrointestinal tract, and are particularly abundant in the infant gut. They are believed to exert various health-promoting effects^1,2^, although little is known about the precise mode of action behind such activities. This knowledge gap has prompted research efforts, in particular focused on the comparative and functional genomics of bifidobacteria^3–5^. Currently, a total of 420 bifidobacterial genome sequences are publicly available (November 2017, https://www.ncbi.nlm.nih.gov/genome), that have, combined with functional genomics data, provided important insights into the molecular mechanisms of adaptation of these bacteria to the gut environment, in particular related to their ability to metabolize dietary and host-derived carbohydrates^6,7^. Autochthonous human gut bifidobacteria have been reported to possess the ability to utilize particular carbohydrates that are not digested by the host, such as human milk oligosaccharides^8^ and a variety of plant-derived sugars^9^. Bifidobacteria employ a very specific carbohydrate assimilatory pathway, commonly referred to as the bifid shunt, which can produce 2.5 molecules of ATP per molecule of glucose as opposed to the hexose monophosphate pathway which generates just 2 ATP/glucose^10^. The superior energy output of the bifid shunt, together with the observed versatility in carbohydrate assimilation abilities are likely to contribute to the success by which bifidobacteria form stable and dominant populations in the gastro intestinal tract^11–13^. Despite the differences in prevalence of each bifidobacterial species with ageing, *Bifidobacterium longum* subsp. *longum* appears to be widely distributed in the gut of adult and elderly subjects, as well as in the infant gut^14,15^. The broad ecological fitness of members of this subspecies is also supported by a recent study on *B. longum* subsp. *longum* AH1206. After oral administration this particular strain was shown to persist in the gut of a sizeable proportion of healthy human subjects for at least 6 months^16^.

Comparative analysis of *B. longum* genomes has revealed the abundant presence of genes related to carbohydrate metabolism, reflecting ecological specialization of three of the currently recognized four subspecies, *B. longum* subsp. *infantis*, *longum* and *suis* (Yanokura *et al*.^17^). In addition, the majority of strain-specific genes or loci in this species (as based on COG analysis) include predicted transposons and phage-derived regions, episomes, as well as genes involved in biosynthesis of cell envelope polymers, fimbriae and R/M systems^18,19^. Furthermore, the existence of a fifth subspecies in this taxon has recently been proposed, based on phylogenetic analysis of the *B. longum* core genome^19^, thus showing the necessity of performing whole genome sequencing to obtain a clear taxonomical classification of members of this species.

The above mentioned studies have provided new insights into the genomic diversity of this species, although we currently do not know if there are specific genetic adaptations that allow *B. longum* subsp. *longum* to be present in either infant, adult and/or elderly hosts. The aim of the present study was therefore to examine genomic differences between a collection of *B. longum* subsp. *longum* strains that had been isolated from human hosts of varying ages.

## Methods

### Microbiota analysis

A total of 453 fecal samples (Table 1, Supplementary Table S1), stored at −80 °C, were obtained as part of a previously described cross-sectional study^13^. The study was approved by the Local Ethics Committee of the non-profit organization Japan Health Promotion Supporting Network (Wakayama, Japan) and the ethics committee of Kensyou-kai Incorporated Medical Institution (Osaka, Japan). Written informed consent was obtained from all subjects or their legal guardians or relatives. All methods were performed in accordance with the relevant guidelines and regulations. Fecal DNA isolation, subsequent (partial) 16 S rRNA gene sequencing using an Illumina MiSeq, and instrument quality filtering of the sequence data in fastq format were performed as described previously^13^. Briefly, 20 mg of a fecal sample was suspended in 450 μl of extraction buffer (100 mM Tris-HCl, 40 mM ethylene diamine tetra acetic acid, pH 9.0) and 50 μl of 10% sodium dodecyl sulfate. Glass beads (300 mg; diameter, 0·1 mm), and 500 μl of buffer-saturated phenol was then added to the suspension followed by vigorous mixing of the sample for 30 s using a FastPrepTM FP 100 A (Funakoshi, Tokyo, Japan) at a power level of 5.0. After centrifugation at 14,000 x g for 5 min, 400 μl of supernatant was extracted with phenol-chloroform and DNA from 250 μl of the resulting supernatant was then precipitated with an equal volume of isopropanol. The V3-V4 region of the bacterial 16 S rRNA gene was amplified using the primer pair Tru357F (5′-CGCTCTTCCGATCTCTGTACGGRAGGCAGCAG-3′) and Tru806R (5′-CGCTCTTCCGATCTGACGGACTACHVGGGTWTCTAAT-3′). A 1-μl sample of the PCR product was then amplified using the following barcoded primers adapted for the Illumina MiSeq: Fwd 5′-AATGATACGGCGACCACCGAGATCTACAC XXXXXXXX ACACTCTTTCCCTACACGACGCTCTTCCGATCTCTG-3′, Rev 5′-CAAGCAGAAGACGGCATACGAGAT XXXXXXXX GTGACTGGAGTTCAGACGTGTGCTCTTCCGATCTGAC-3′, where X represents a barcode base. Potential chimeric sequences were removed using UCHIME, assigned to operational taxonomic units (OTUs) using open-reference OTU picking with a 99% threshold of pairwise identity, and then classified taxonomically using BLASTN against the NCBI non-redundant database. In this manner OTUs were assigned to a particular species, rather than subspecies (16 S rRNA sequencing does not allow accurate assignment at subspecies level). Hierarchical clustering analysis was performed using MeV suite Version 4.9 (https://sourceforge.net/projects/mev-tm4/) using calculated distances based on Pearson correlation.

**Table 1:** Sample distribution.

age segmented group		subject number (Male/Female)			
Segmentation	(mean ± sd)	for microbiota analysis		for genome analysis	
Preweaning	(0.3 ± 0.1)	13	(7/6)	5	(3/2)
Weaning	(0.8 ± 0.4)	12	(8/4)	5	(3/2)
weaned-3	(2.4 ± 0.6)	22	(11/11)	11	(6/5)
4–9 years old	(5.9 ± 1.8)	17	(7/10)	7	(4/3)
10–19 years old	(14.1 ± 3.6)	10	(7/3)	9	(6/3)
20–29 years old	(25.8 ± 2.6)	42	(14/28)	7	(2/5)
30–39 years old	(34.3 ± 2.6)	117	(56/61)	12	(6/6)
40–49 years old	(43.4 ± 3.1)	37	(14/23)	9	(5/4)
50–59 years old	(53.7 ± 2.8)	34	(14/20)	10	(5/5)
60–69 years old	(64.2 ± 2.9)	42	(14/28)	10	(2/8)
70–79 years old	(75.5 ± 2.9)	31	(12/19)	11	(4/7)
80–89 years old	(83.2 ± 2.4)	51	(17/34)	12	(7/5)
90–99 years old	(94.2 ± 2.7)	19	(4/15)	5	(4/1)
100–104 years old	(101.3 ± 1.8)	6	(0/6)	0	
Sum		453	(185/268)	113	(57/56)

Gut microbiota were analyzed for one sample per subject. Per fecal sample a single *B. longum* subsp. *longum* isolate was selected, except for isolates MCC10031 and MCC10212, which both originated from a 33 year old subject.

### Isolation of strains

Three hundred ninety three of the 453 assessed fecal samples (nearly 87%) were confirmed to contain *B. longum* subsp. *longum* DNA employing PCR and a subspecies-specific primer pair^20^, of which 177 were selected to retrieve bifidobacteria by cultivation on TOS propionate agar (Eiken Chemical, Tokyo, Japan) supplemented with 50 mg/l mupirocin (Merck KGaA, Darmstadt, Germany). Following incubation at 37 °C for 48 hours under anaerobic conditions, ten colonies per sample were randomly selected and were examined by colony PCR using the above mentioned species-specific primer set. Each identified *B. longum* subsp. *longum* isolate was cultivated in Difco Lactobacilli MRS (Becton Dickinson, NJ) supplemented with 0.05% L-cysteine HCl (Kanto Chemical, Tokyo, Japan) at 37 °C for 16 hours under anaerobic conditions before DNA extraction. In this manner a total of 162 *B. longum* subsp. *longum* strains were isolated, of which a proportion (based on age representation) was selected for sequencing (see below).

### Genome sequencing and data assembly

Genomic DNA was extracted using the DNeasy Blood & Tissue Kit (Qiagen, Valencia, CA) according to the manufacturer’s protocol. Library construction was performed as previously described^21^. Paired-end sequencing was performed using an Illumina MiSeq instrument with the MiSeq v3 Reagent Kit (Illumina Inc., San Diego, CA, USA), achieving a coverage of at least 30-fold. Quality trimming and *de novo* assembly of raw reads was performed using the CLC Genomics Workbench (v 8.0) software package (Qiagen, Valencia, CA) with default settings, except for contig length (minimum contig length = 500 bp). Contigs composed of less than 100 reads were removed.

### General features prediction

Open Reading Frame (ORF) prediction was performed using PRODIGAL (version 2.6; http:// prodigal.ornl.gov/) and supported by BLASTX v2.2.26^22^ alignments (including the genomes retrieved from NCBI). Automatic annotation was performed based on BLASTP v2.2.26 analysis using *B. longum* subsp. *longum* NCC2705 as reference (NCBI Reference Sequence: NC_004307.2) and the non-redundant protein database curated by the National Centre for Biotechnology (http://www.ncbi.nlm.nih.gov/). Where necessary, manual editing was performed using Artemis v.15^23^ which was employed for output visualization. Where relevant, annotations were further refined using a combination of protein family (Pfam)^24^ and COG^25^ databases. Transfer RNA genes were identified using tRNAscan-SE v1.4^26^. The presence of a signal peptide in deduced proteins was predicted using the SignalP 4.1 server (http://www.cbs.dtu.dk/services/SignalP/).

### Comparative genomics

A total of 32 *B. longum* genomes were retrieved from the NCBI public database for comparative purposes (Supplementary Table S2). Amino acid sequence comparisons were performed using an all-against-all, bi-directional BLAST alignment (cut-off: E-value 0.0001, with at least 50% identity across at least 50% of either protein sequence), followed by clustering into protein families using the Markov Cluster Algorithm (MCL) implemented in the mclblastline pipeline v12-0678^27^. Gene families (abbreviated here as GF) were divided into two groups, the core genes, being present in all strains, and the dispensable genes, being present in a subset of the investigated strains. Distances were based on the covariance value and were calculated for the hierarchical clustering analysis. Genomic alignments were conducted using the progressive Mauve tool with default settings^28^.

### Classification of genes enriched in B. longum subsp. longum strains isolated from either younger or older subjects

A total of 1,538 dispensable GF, of which each was present in between 6 and 107 strains (of a total number of 113 sequenced strains, here named MCC strains), was employed for the evaluation of genes enriched in strains from subjects based on their age. After calculating the detection rate of dispensable GF in genomes grouped by age segments, hierarchical clustering analysis was performed using the distances based on the Pearson correlation.

### Strain-specific PCR

After detection of clustered regularly interspaced short palindromic repeats (CRISPR)-*cas* systems by CRISPR finder in our dataset (http://crispr.i2bc.paris-saclay.fr/Server/), a specific primer-pair was designed for each strain based on the identified protospacers (Supplementary Table S3). The specificity of the designed primers was confirmed by both BLASTN alignments performed against all genomes in this study, and PCR assays using DNA samples extracted from all MCC strains, *B. longum* subsp. *longum* JCM 1217 and two randomly selected fecal samples as negative controls (Supplementary Table S4). Each 1 μl sample of fecal DNA, at a concentration of approximately 10 to 200 ng/μl as measured using a Nanodrop 2000 (Thermo Fisher Scientific, Waltham, MA, USA), was used as a template for amplification using the following protocol: preheating at 94 °C for 3 min; 30 cycles of denaturation at 94 °C for 20 s, annealing at the appropriate temperature (see Supplementary Table S3) for 20 s and extension at 72 °C for 20 s; and a final terminal extension at 72 °C for 5 min. The size of amplified DNAs was confirmed using the QIAxcel system (Qiagen, Valencia, CA, USA).

### Phylogenetic analysis

Phylogenomic inference was performed based on the alignment of a set of single core GF defined by MCL clustering (Supplementary Table S5). As for a phylogenetic tree based on the strains isolated from members of family, *B. longum* subsp. *longum* JCM 1217 ^T^ was used as an outgroup. Each GF was aligned using the MUSCLE alignment tool^29^. Phylogenetic trees were computed using the maximum-likelihood in PhyML v3.0^30^ and concatenated with statistical assessment based on 100 bootstrap replicates; the resulting consensus tree was computed using the Consense module from the Phylip package v3.69 using the majority rule method (http://evolution.genetics.washington.edu/phylip.html).

### Statistical analysis

All analyses without calculation of a false discovery rate (FDR) were performed using SPSS version 22.0 statistical software (IBM Corp., Armonk, NY, USA). Intergroup differences were analysed using Mann-Whitney U test, followed by FDR calculation on R software (ver. 3.3.0) with the qvalue package (http://www.bioconductor.org/packages/release/bioc/html/qvalue.html) (n = 1,538). Correlation analysis was performed by Spearman’s correlation coefficient (n = 113). For all statements, p-values of < 0.05 or FDRs of < 0.2 were considered statistically significant.

### Data Availability

DNA sequences corresponding to 16 S rRNA gene data were deposited in DDBJ under accession numbers DRA004160 and DRA005774 (Supplementary Table S1). Genome sequences of *B. longum* subsp. *longum* were submitted to GenBank (accession numbers are listed in Supplementary Table S6).

## Results

### Gut microbiota composition

**Figure 1:**
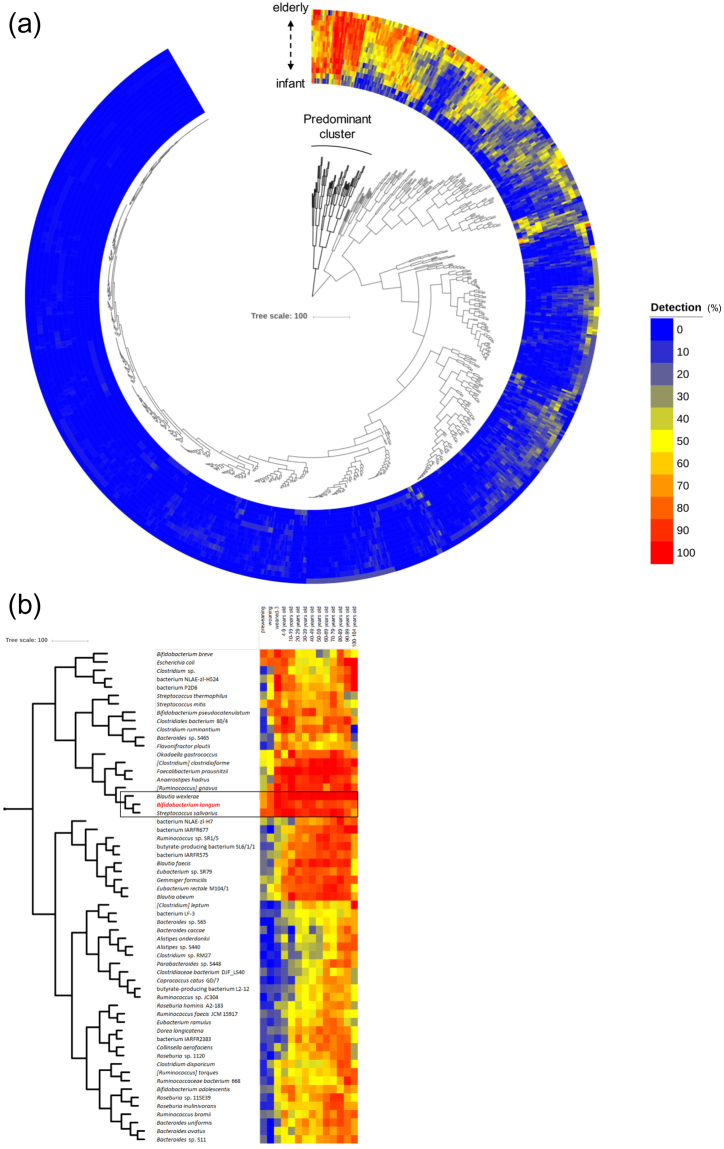
Distribution of gut microbiota in Japanese subjects. (**a**) HCL clustering based on the detection rate of 871 OTUs at species level found in faecal samples of 453 healthy Japanese subjects between the ages of 0 and 104 years. The Heat map displays the detection rate in each age-segmented group (see Table 1) from infants to elderly ordered from inner to outer circles. A predominant cluster of OTUs determined by HCL clustering and composed of 59 species is also indicated. (**b**) Enlarged view of the predominant cluster is depicted in Fig. 1a. The three species showing the highest prevalence rate (>50%) in each segmented age are enclosed by a solid line

Following sequencing of the amplified V3-4 region of the 16 S rRNA gene, a total of 871 different OTUs at species level were detected in faecal samples from 453 healthy Japanese subjects aged between 0 and 104 years (Table 1 & Fig. 1a), with 5,366 ± 5,129 high quality reads per sample (Supplementary Table S1). Based on the obtained species-level gut microbiota composition of these Japanese subjects (Fig. 1a), only three species (*Blautia wexlerae*, *Streptococcus salivarius* and *Bifidobacterium longum*) were detected in more than 50% of the subjects across the segmented age groups (Fig. 1b). This result highlights the broad distribution of *B. longum* across (healthy) human lifespan as a distinctive property among the hundreds of bacterial species that are present in the healthy human gut.

### Isolation of strains

In order to investigate possible genotypical reasons for the broad distribution of *B. longum*, we decided to assess the genome diversity of *B. longum* strains present in the cohort under study. A PCR-based approach employing a subspecies-specific primer pair showed that 393 out of 453 tested fecal samples contained *B. longum* subsp. *longum* (nearly 87%). A total of 162 *B. longum* subsp. *longum* strains were then isolated from the fecal samples of 177 healthy Japanese subjects (87 male, 90 female; aged between 0 and 98 years), which were randomly selected, yet in a way that all age segments were uniformly represented. We then selected between 5 and 12 strains from each segmented-age group for genome sequence analysis (Table 1). In this manner, genome sequences were generated for 113 strains (Supplementary Table S1). A single *B. longum* subsp. *longum* isolate was selected per fecal sample, except for isolates MCC10031 and MCC10212, which both originated from a 33 year old subject. Taxonomical classification of all 113 strains was confirmed by phylogenetic analysis performed on the *B. longum* core genome (Supplementary Fig. S1).

### General features of *B. longum* subsp. *longum* genomes

Salient features of each of the newly determined *B. longum* subsp. *longum* genomes are presented in Supplementary Table S6. Sequencing generated between 16 and 96 contigs for each strain with a minimum sequence coverage of 30.5 fold. The predicted genome size ranged between 2.19 Mb and 2.63 Mb, possessing an average G + C% content of 60.0%, and an average predicted ORF number of 2,028 per genome. The number of predicted tRNA genes ranged between 50 to 64 in the majority of strains. However, 74 or more tRNA genes were identified in 15 strains, which were all shown to harbour a predicted megaplasmid which was shown to contain between 14 and 26 tRNA genes. This is consistent with what has previously been determined for the *B. breve* megaplasmid pMP7017, which carries a set of 14 tRNA genes^31^, and enforces the notion that the carriage of multiple tRNA genes provides translational support to the host and constitutes a common feature of these mobile elements in (bifido)bacteria^31,32^. The Cluster of Orthologous Group (COG) classification performed for the identified ORFs shows that the highest proportion of identified genes encode proteins that are predicted to be involved in general cellular housekeeping functions, especially those related to carbohydrate (12.13%) and amino acid (11.75%) metabolism, and associated transport activities (Supplementary Fig. S2), being consistent with previous reports^18,19^.

### Comparative genomics

**Figure 2:**
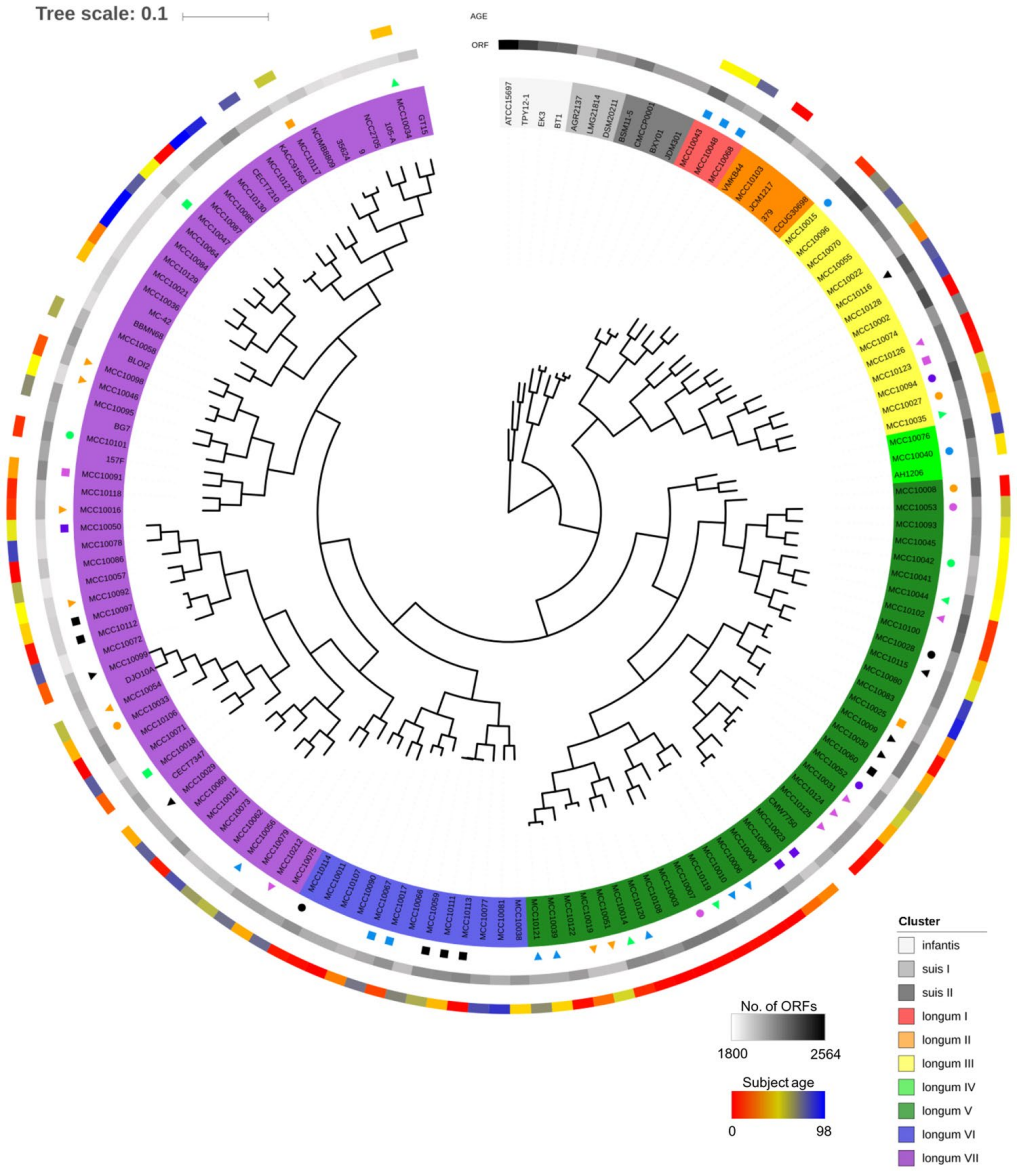
MCL clustering of *B. longum* strains. MCL clustering based on the open reading frame (ORF) content of 113 novel *B. longum* subsp. *longum* isolates obtained from healthy Japanese subjects (age range between 0 and 98 years), plus those of 32 publicly available *B. longum* representatives. Strain names are colour-coded based on the MCL computed clusters. The circles, from outside to inside, indicate subject age, number of ORFs per genome and, where applicable, family origin of strains (as indicated by similar symbols).

MCL clustering performed on the deduced proteins from the identified ORFs of the 113 genome sequences obtained in the current study combined with 32 publicly available *B. longum* genomes showed that each *B. longum* subspecies is clearly delineated as based on presence/absence of gene families, and that all strains sequenced in the current study (designated MCC strains) belong to *B. longum* subsp. *longum* (Fig. 2). *B. longum* subsp. *longum* strains were shown to separate into seven clusters, where the majority of cluster-specific genes or loci represent hypothetical proteins predicted to be a part of a prophage, an integrated episome or a megaplasmid (Supplementary Fig. S3). Notably, clear homologs of the conjugative megaplasmid pMP7017 from *B. breve* JCM7017^31^ were identified in fifteen MCC *B. longum* strains. Fourteen of these megaplasmid sequences contained the homologous region of two adjacent clustered regularly interspaced short palindromic repeats (CRISPR)-cas systems (I-E subtype^33^) present on megaplasmid pMP7017 (Supplementary Fig. S4). Further assessment of the two adjacent CRISPR-Cas systems showed the presence of a varying number of different protospacer sequences (Supplementary Table S7), of which 10.7% and 2.9% correspond to previously identified bifidobacterial prophages and plasmids, respectively. We also identified CRISPR-Cas systems on the chromosome of forty of our isolated strains. These forty CRISPR-Cas systems were shown to be representatives of the II-C subtype without tracrRNA except for strain MCC10117, which was shown to encode a I-U subtype CRISPR-Cas system (Supplementary Fig. S4, Supplementary Table S7). The number of spacers ranged between 24 and 58, of which 73.8% correspond to publicly available bifidobacterial genome sequences by BLAST analysis. Our all-against-all comparison of spacer sequences indicated that the CRISPR-Cas system on the megaplasmids exhibited more variation than those present on the chromosomes, which may be an indication of their activity (supplementary Table S7).

### Genes enriched in B. longum subsp. longum strains isolated from either younger or older subjects

**Figure 3:**
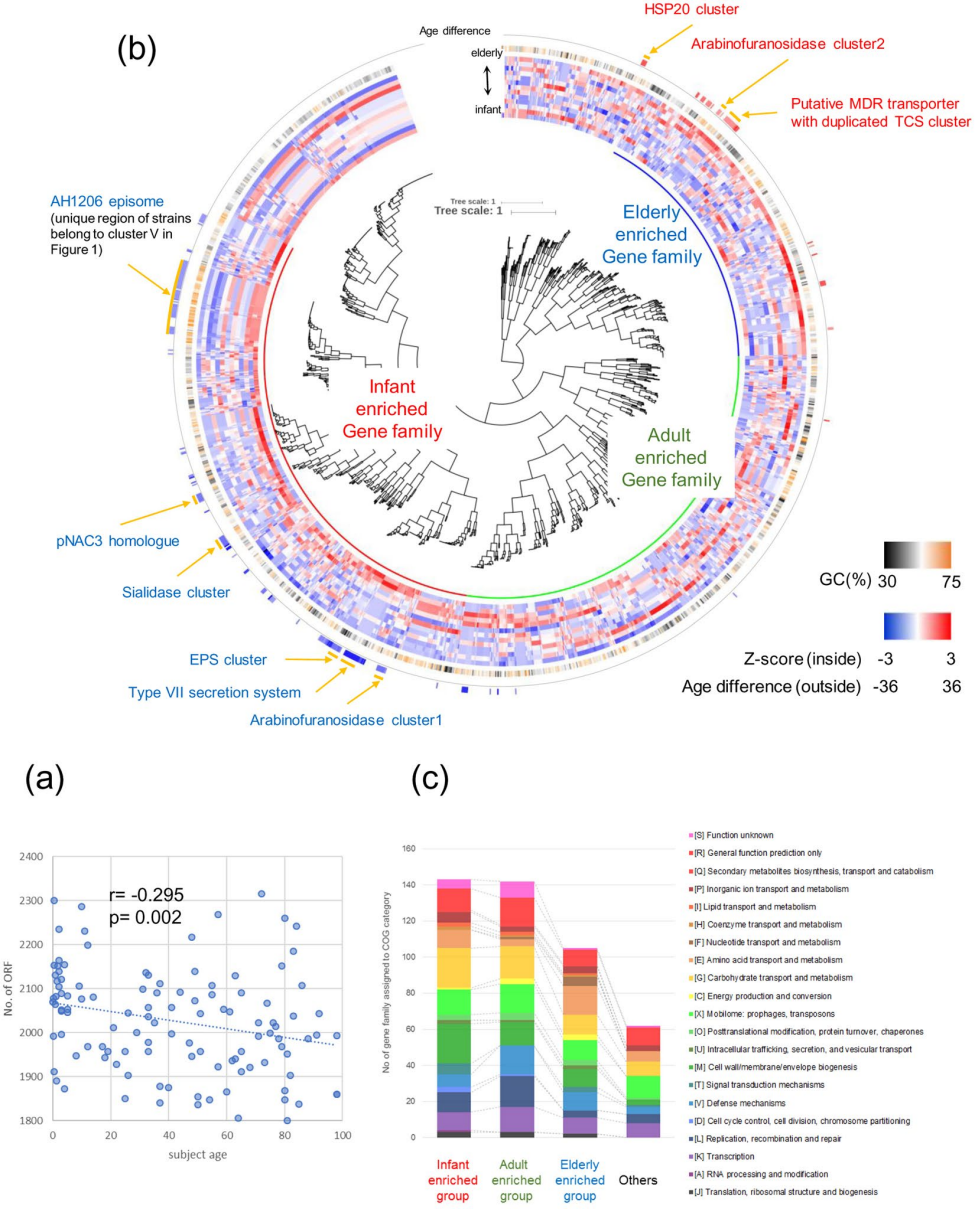
Genes enriched in *B. longum* subsp. *longum* strains isolated from subjects of varying age. (**a**) Correlation analysis with relative trendline computed between ORF number of *B. longum* subsp. *longum* strains and age of subject from whom each strain was isolated. (**b**) HCL clustering based on the detection rate of gene families across each age segmented group. From outer to inner circle a circular heatmap represents gene families significantly enriched in strains isolated from younger or older subjects, G + C % of the gene family and the computed detection rate z-scores of gene families in each age segmented group. (**c**) Bar chart showing the COG classification of genes detected in each age cluster in panel b.

**Figure 4:**
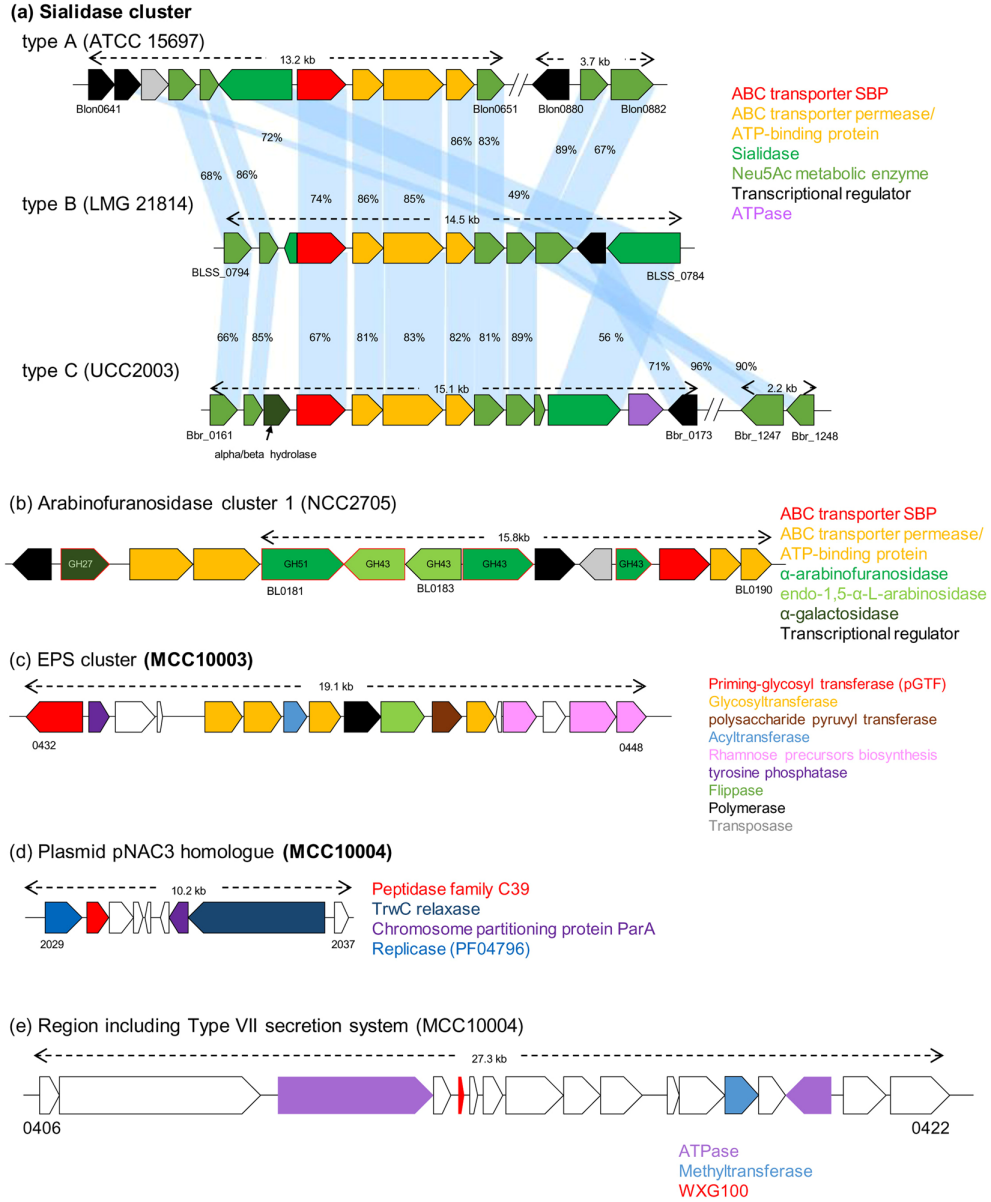
Gene clusters enriched in younger subjects. Locus maps display the genetic organization of clusters enriched in younger subjects. Reference strain name is indicated in parenthesis and genes are colored based on predicted function (uncolored genes represent hypothetical proteins). (**a**) Comparison of three types of sialidase clusters observed in our dataset with the corresponding reference strain indicated. (**b**) Locus map of the arabinofuranosidase cluster 1 detected in our dataset. Red outer boundary indicates the glycoside hydrolase predicted as extracellular. (**c**) Locus map of the exopolysaccharide biosynthesis cluster where homologues are located on the same contig in five out of 17 strains. (**d**) Plasmid pNAC3 homologue and (**e**) Region encoding a predicted Type VII secretion system.

No correlation was obvious from the MCL hierarchical clustering between isolated strains and subject age (Fig. 2), although a significant negative correlation exists between the number of ORFs of a given *B. longum* subsp. *longum* strain and the age of the subject from which the strain had been isolated (Fig. 3a). This suggests that *B. longum* subsp. *longum* strains isolated from younger subjects possess on average a higher number of genes as compared to strains isolated from an older subject. We therefore decided to perform an in depth investigation to identify genes (or gene families, GF, see Materials and Methods) that are differentially associated with *B. longum* subsp. *longum* strains isolated from younger and/or older subjects. Interestingly, this analysis allowed us to classify GF into infant, adult and elderly-enriched groups, as well as genes that exhibit no relationship with subject age (Fig. 3b). In accordance with the correlation shown in Fig. 3a, the number of GF with a COG category assignment was also lower in the elderly-enriched GF set as compared to those found in corresponding infant- and adult-enriched GF (Fig. 3c). Furthermore, the number of (dispensable) GF classified as being involved in carbohydrate transport and metabolism in the infant-enriched group was twice that in the elderly-enriched group (22 vs. 11), whereas the number of GF associated with defense mechanisms, transcription and replication, recombination and repair was the highest in the adult-enriched group. Employing the Mann-Whitney U test showed that 169 GF were significantly enriched in *B. longum* subsp. *longum* strains isolated from younger subjects (Fig. 3b, Supplementary Table S8), whereas 55 GF were enriched in strains isolated from older subjects (Fig. 3b, Supplementary Table S9). The significantly enriched genes in younger subjects included a sialidase-encoding cluster, an α-arabinofuranosidase gene cluster, pNAC3 (a 10 kb plasmid) homologue, capsule biosynthesis related genes and a Type VII secretion system, as well as some prophage regions also found in the AH1206 episome^16^ (contributing to cluster V in Fig. 2; Fig. 4 and Supplementary Table S8).

It may not be surprising that the sialidase clusters are enriched in the infant-enriched functions considering that human milk oligosaccharides are commonly decorated with sialic acid, such as in the case of sialyllactose and mono/di-sialyl lacto-N-tetraose^34^. In fact, 10 strains of *B. longum* subsp. *longum* were found to contain genes homologous to those of *B. longum* subsp. *infantis* ATCC 15697 (Blon0641-0651 and Blon0880-0885^35^; this genetic constellation is designated here as type A), whereas the genome of nine *B. longum* subsp. *longum* strains harbour a similarly organized sialidase-related cluster as that present in *B. longum* subsp. *suis* LMG 21814 (BLSS_0784-0794, designated here as type B). The only exception is strain MCC10016 which possesses a sialidase cluster homologous to that of *B. breve* UCC2003 (Bbr_0161-0173 and Bbr_1247-1248^36^, designated here as Type C). The similarity levels among the strains that carry any of these three sialidase clusters are listed in Supplementary Table S10. It is worth mentioning that these three gene cluster types for sialic acid metabolism are all predicted to encode an intracellular sialidase (protein sequence similarity ranging between 49 to 56%).

**Figure 5:**
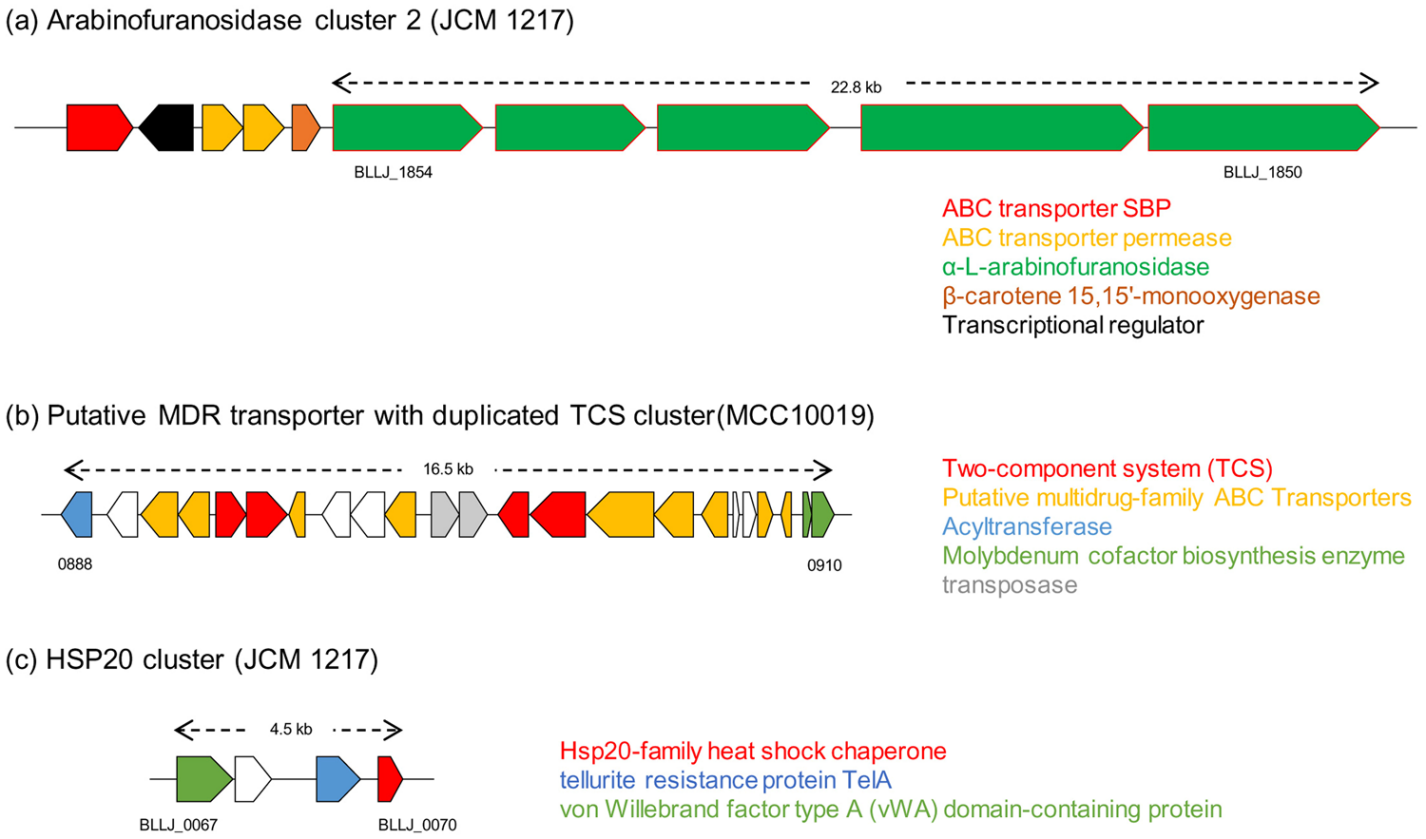
Gene clusters enriched in elderly subjects. Locus maps showing the genetic organization of clusters enriched in elderly subjects. Reference strain name is indicated in parenthesis and genes are colored based on the predicted functions (uncolored genes are predicted to encode hypothetical proteins). (**a**) Locus map of the arabinofuranosidase cluster 2. All glycoside hydrolases shown as red outer boundary are predicted to be extracellular enzymes. (**b**) Putative MDR transporter with duplicated TCS cluster and (**c**) HSP20 cluster.

Genes enriched in older subjects include a cluster of five tandemly arrayed genes that are all predicted to encode extracellular α-L-arabinofuranosidases (BLLJ_1850 to BLLJ_1854 homologues) as well as genes encoding a putative multidrug-family ABC transporter with an associated two-component system (TCS), a genetic cluster containing an Hsp20-family heat shock chaperone, and various prophage regions (Fig. 5, Supplementary Table S9).

### Assessment of possible strain transmission among family members

**Figure 6:**
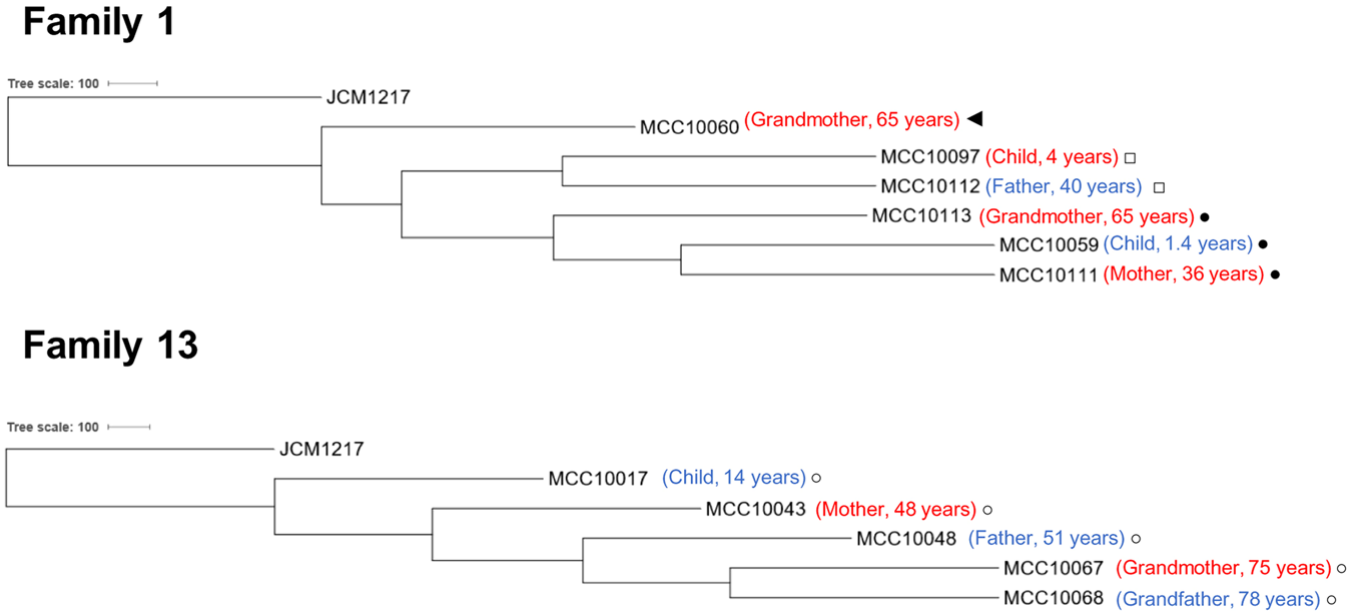
Cladogram of strains transmitted across three generations. Phylogenetic tree representing the relationships between five strains putatively transmitted across three generations. The tree was calculated based on the nucleotide sequence of 642 single-core gene families among 145 *B. longum* strains analysed in this study (see Supplementary Table S5). *B. longum* subsp. *longum* JCM 1217 ^T^ was used as an outgroup. Isolation source for each strain is shown in parenthesis (blue: male; red: female). Same symbols indicate strains predicted to be transmitted between family members.

In seven distinct cases, two strains which had been isolated from two different, but related subjects, were shown to contain a near identical gene content, while in three cases this situation applied to three strains, each of which had been isolated from a different member of the same family (Fig. 2; Supplementary Table S11). Each strain pair found in Fig. 2 was shown to display a very high level of sequence identity including the protospacers of CRISPR-Cas systems, where present (Supplementary Fig. S5). Five pairs of these strains possess a CRISPR-Cas system with fully matching protospacers, whereas in four strain pairs, the region of protospacers in CRISPR-Cas system were partially different (Supplementary Fig. S5, Supplementary Table S7). These findings suggest that such strains had been transmitted between family members, in this case not only between mother-child, which has been reported previously^37^, but also between father and infant, and across three generations. In the latter case, strains MCC10059, MCC10111 and MCC10113 had been isolated from subjects aged 1, 36 and 65 years, whose relationship were grandmother, mother and infant (family 1), and with corresponding ANI values in excess of 99.99%. In another case (family 13), strains MCC10017, MCC10043, MCC10048, MCC10067 and MCC10068 had been isolated from subjects aged 14, 48, 51, 75 and 78 years, whose relationship were adolescent male, mother, father, grandmother and grandfather, and with corresponding ANI values in excess of 99.5% although they are separeted into two groups based on gene content (Fig. 2). Twenty nine of thirty protospacers in the CRISPR-Cas system were commonly present in each of these five strains except for MCC10043, where some of the protospacers seem to have been deleted (Supplementary Fig. S5, Supplementary Table S7). A phylogenetic tree based on the strains isolated from members of family 13 implies that the direction of their transmission had been from older to younger family members or vice versa (Fig. 6).

Furthermore, we evaluated other possible transmission events within family members by strain-specific PCR assay of 86 DNA faecal samples, obtained from subjects with at least one other relative in the study group. A total of 21 strain-specific primer pairs were thus designed for strains possessing a CRISPR-Cas system, based on protospacer-sequences (Supplementary Tables S3 and S4). The obtained results imply that 9 additional strains had been subject to transmission in 7 families (Supplementary Table S11). Surprisingly, some strains were shown to be present in all members (enrolled in this study) of a given family (family 3, 13, 15, 19 and 20). In total, in 12 out of the 22 families involved in this study possible transmission was detected corresponding to 15 cases and involving either two or more strains.

## Discussion

Gut microbiota composition changes with age, and alterations are thought to influence health. Recent studies have shed light on the changes in human gut microbiota composition^13,38^, although the underlying mechanisms that drive such changes are still poorly understood. In the present study, we performed comparative genome analysis aimed at determining the genetic differences of *B. longum* subsp. *longum* strains isolated from human hosts of different ages.

Our findings revealed that infant-associated strains possess a higher number of ORFs, some of which are related to carbohydrate utilization including sialic acid metabolism, which might be the result of an adaptive strategy of *B. longum* to survive the infant gut environment, characterized by a high amount of nutrients derived from mother’s milk. It has previously been shown that sialylated milk oligosaccharides promote microbiota-dependent growth in animal models of undernurished infants^39^, suggesting that these gene clusters involved in the sialic acid metabolism play an important role in sustaining healthy infant growth and development. Furthermore, our analysis indicates that homologues of plasmid pNAC3 and genes encoding a type VII secretion system are also enriched in infant strains. Plasmid pNAC3 encodes a peptidase of the C39 family, representatives of which are known to act as bacteriocin-processing endopeptidases^40^. This finding is consistent with a report which annotated one of the ORFs on this plasmid as a putative bacteriocin^41^. Type VII secretion systems play varying roles in virulence, sporulation and translocation of various components^42^, and are present throughout the phylum Actinobacteria^43^. A recent report suggests that such a secretion system specified by *Staphylococcus aureus* secretes a nuclease toxin that targets competitor bacteria (Cao *et al*.^44^). The significance of the enrichment of these genes in infant isolates remains unclear, though a distict possibility is that they contribute to successful colonization and persistence of *B. longum* in the infant gut via suppressing growth of other bacteria.

In elderly-associated strains, we observed the enrichment of a unique cluster with five tandemly arrayed genes that are predicted to encode extracellular α-L-arabinofuranosidases, which are known to be involved in hydrolysis of arabinogalactan, arabinoxylan or arabinan in concert with one or more extracellular galactanases^9^. Another α-L-arabinofuranosidase-encoding cluster was enriched in infant-derived strains, however, this cluster contains only one out of the five extracellular arabinosidase-encoding genes. According to a National Health and Nutrition Survey 2015, elderly Japanese individuals eat much more vegetables than their younger counterparts (http://www.mhlw.go.jp/stf/houdou/0000142359.html). The enrichment of the α-L-arabinofuranosidase-encoding cluster may therefore be an advantage for *B. longum* subsp. *longum* strains in terms of enhanced adaptation and increased competitiveness in the elderly gut environment as this genetic trait allows them to efficiently cleave and incorporate arabino-mono and/or oligosaccharide from foods containing insoluble fibers that are rich in arabinogalactan, arabinoxylan and/or arabinan.

We also observed two clusters related to environmental response among GF enriched in elderly subject. Of these, the homologue of Hsp20-family heat shock chaperone has been reported to be induced by severe heat shock and osmotic shock in *B. breve* UCC2003^45^. This protein was flanked by a tellurite resistance protein and a von Willebrand factor type A (vWA) domain-containing protein which mediates adhesion via metal ion-dependent adhesion sites^46^. It is reasonable to argue that strains endowed with these abilities possess enhanced adaptive and survival traits.

*Bifidobacterium* species have been reported to exhibit a rather high frequency of CRISPR-Cas occurrence (77%)^33^. In the current study, we found three different CRISPR-Cas subtypes in 44.2% (50 of 113) of the analysed *B. longum* subsp. *longum* strains. Of these the most frequently observed subtype was II-C, which previously has been reported to commonly contain tracrRNA genes^47^. However, no tracrRNA was found in the II-C subtype members of our study in accordance with a previous study on *B. longum* subsp. *longum* DJO10A^47^. Further research, such as gene expression assays, will be needed to determine if these systems are functionally fully active. Nonetheless, given the presence of the various protospacers in each strain, it is conceivable that these CRISPR-Cas systems constitute an active barrier against invading DNA.

While different individuals may share a core microbial species (as shown in Fig. 1), it has been shown that these common species basically consist of distinct strains^12,48^. However, vertical microbiome transmission from mothers to their infants at strain level was suggested by a metagenomic approach^49^, while other studies have provided compelling evidence for the occurrence of vertical transmission of bifidobacterial strains from mother to newborn^50–52^. Milani *et al*. indicated the persistence of two strains transmitted from mother to infant at 6 months by PCR analyses targeting unique genes^52^. Chiaplin *et al*. suggested a long-term persistence (up to 10 years) of *B. longum* subsp. *longum* in a child as demonstrated by PAGE analysis of PCR-amplified variable number tandem repeat (VNTR) loci^18^. Asnicar *et al*. have shown that transmitted bifidobacterial strains are transcriptionally active in the guts of both adult and infant based on faecal sample analysis^49^. However, the current study is the first report on the identification of a particular strain that appears to have been transmitted across three generations based on the high similarity of their genome sequences. Furthermore, it is noteworthy that we not only observed transmission events between mother and child, but also between father and child or between husband and wife. In total, twenty-five out of sixty-three strains obtained from subjects with at least one other relative in the study group appears to have been transmitted between family members. Transmission is further substantiated by the finding that such transmitted strains possess an identical CRISPR locus with the same order of protospacers. Even though we currently do not know the transfer directionality and specific transfer route of these bifidobacterial strains (which could be either vertical or horizontal), our findings clearly show that person-to-person transmission of *B. longum* subsp. *longum* is a very common phenomenon within families. At birth the essentially empty niche of the neonatal gut may freely facilitate colonization of individual strains acquired from family members, though these strains have to be able to compete in an environment where human milk may be the sole nutrition. During weaning when diet will be subject to significant changes, the composition of the resident microbiota and persistence of *B. longum* strains are challenged by novel growth substrates and therefore newly colonizing and competing bacteria. Furthermore, it is known that *B. longum* subsp. *longum* is capable of taking up (long-term) residence in a human host provided that the composition of the resident bacterial species coupled with the carbohydrate utilization capabilities of that strain facilitate this^16^. Therefore, our findings argue for a scenario where bifidobacterial strains may join a newly forming or resident gut microbiota at any point during the life time of the host, though presumably with a higher chance of transfer at the early stages of life. Later in life common dietary sources or environmental exchanges may constitute possible vehicles and routes for strain transmission, although the precise mechanistic details of such transfer will require further research. Interestingly, some of the species that are prevalent in the gut (e.g. *B. breve*, *Streptococcus salivarius* and *B. longum*; Fig. 1b) are also used as functional ingredients in particular foods, and this raises the possibility that diet is a major source of strains that populate the gut. However, none of the *B. longum* subsp. *longum* genomic sequences we identified as possibly transmitted between family members (see Supplementary Fig. S5) correspond to strains contained in commercially available products (where such genomic data was available, data not shown). Another strain transfer possibility is via an environmental reservoir, such as the family home. Gilbert *et al*. have reported that each home has its own microbiome, where this home microbiome is largely being sourced from humans^53^. Furthermore, a bacterial ecology study of house dust found that certain bacterial taxa of this niche are also members of the gut microbiota^54^. These findings imply that transfer of *B. longum* subsp. *longum* strains may indeed commonly occur with the family home, although precise details of such presumptive strain transmission are currently not known. Further research will therefore need to be performed to accurately determine the extent, location and timing of such transmission events, and to determine if other (bifido)bacterial members of the human microbiota possess similar versatile transmission, colonization and persistence abilities.

In conclusion, we hereby demonstrate that strain-specific differences observed between *B. longum* subsp. *longum* genomes isolated from infant and elderly subjects can be correlated to their adaptation to particular ecological niches (or age-dependent diets). In addition, we observed that certain strains had most likely been subject to transmission across 2 or even 3 generations. These results suggest that the broad distribution of *B. longum* subsp. *longum* across the human lifespan is due to a high transfer level between family members. We furthermore identified a variety of genes which could enhance their adaptation and increase competitiveness in the gut environment, enriched in genomes corresponding to specific ages.

## Electronic supplementary material

Supplementary Information

Supplementary Tables

## References

[1] Ventura M, Turroni F, Motherway MOC, MacSharry J, van Sinderen D (2012). Host-microbe interactions that facilitate gut colonization by commensal bifidobacteria. Trends Microbiol..

[2] Tojo R (2014). Intestinal microbiota in health and disease: Role of bifidobacteria in gut homeostasis. World J Gastroenterol.

[3] Ventura M (2009). Genome-scale analyses of health-promoting bacteria: probiogenomics. Nat. Rev. Microbiol..

[4] Fukuda S (2011). Bifidobacteria can protect from enteropathogenic infection through production of acetate. Nature.

[5] O’Connell Motherway M (2011). Functional genome analysis of *Bifidobacterium breve* UCC2003 reveals type IVb tight adherence (Tad) pili as an essential and conserved host-colonization factor. Proc. Natl. Acad. Sci. USA.

[6] Callaghan AO, Sinderen D (2016). Van. Bifidobacteria and their role as members of the human gut microbiota. Front. Microbiol..

[7] Milani C (2016). Genomics of the genus *Bifidobacterium* reveals species-specific adaptation to the glycan-rich gut environment. Appl. Environ. Microbiol..

[8] James K (2016). *Bifidobacterium breve* UCC2003 metabolises the human milk oligosaccharides lacto-N-tetraose and lacto-N-neo-tetraose through overlapping, yet distinct pathways. Sci. Rep..

[9] Fujita K, Sakaguchi T, Sakamoto A, Shimokawa M, Kitahara K (2014). *Bifidobacterium longum* subsp. *longum* exo-β-1,3-galactanase is an enzyme for the degradation of type II arabinogalactan. Appl. Environ. Microbiol..

[10] Sela, D. A., Price, N. P. J., and Mills, D. ‘Metabolism of bifidobacteria,’ in Bifidobacteria: Genomics and Molecular Aspects, eds B. Mayo and D. van Sinderen. *Caister Acad. Press* (2010).

[11] Martínez, I., Muller, C. E. & Walter, J. Long-Term Temporal Analysis of the Human Fecal Microbiota Revealed a Stable Core of Dominant Bacterial Species. *PLoS One* **8**, (2013). 10.1371/journal.pone.0069621 PMC3712949 23874976

[12] Schloissnig S (2012). Genomic variation landscape of the human gut microbiome. Nature.

[13] Odamaki T (2016). Age-related changes in gut microbiota composition from newborn to centenarian: a cross-sectional study. BMC Microbiol..

[14] Turroni F (2009). Exploring the diversity of the bifidobacterial population in the human intestinal tract. Applied and environmental microbiology.

[15] Françoise Gavini CC (2001). Differences in the Distribution of Bifidobacterial and Enterobacterial Species in Human Faecal Microflora of Three Different (Children, Adults, Elderly) Age Groups. Microb. Ecol. Health Dis..

[16] Maldonado-Gómez MX (2016). Stable Engraftment of *Bifidobacterium longum* AH1206 in the Human Gut Depends on Individualized Features of the Resident Microbiome. Cell Host Microbe.

[17] Yanokura E (2015). of *Bifidobacterium longum* by multilocus approaches and amplified fragment length polymorphism: Description of *B. longum* subsp. *suillum* subsp. nov., isolated from the faeces of piglets. Syst Appl Microbiol..

[18] Chaplin AV (2015). Intraspecies Genomic Diversity and Long-Term Persistence of *Bifidobacterium longum*. PLoS One.

[19] O’Callaghan A, Bottacini F, O’Connell Motherway M, van Sinderen D (2015). Pangenome analysis of *Bifidobacterium longum* and site-directed mutagenesis through by-pass of restriction-modification systems. BMC Genomics.

[20] Matsuki T (2004). Quantitative PCR with 16S rRNA-gene-targeted species-specific primers for analysis of human intestinal bifidobacteria. Applied and environmental microbiology.

[21] Odamaki, T. *et al*. Comparative Genomics Revealed Genetic Diversity and Species/Strain-Level Differences in Carbohydrate Metabolism of Three Probiotic Bifidobacterial Species. 56780 (2015). 10.1155/2015/567809 PMC4506816 26236711

[22] Altschul SF, Gish W, Miller W, Myers EW, L. D (1990). Basic local alignment search tool. J Mol Biol.

[23] Carver T, Harris SR, Berriman M, Parkhill J, McQuillan JA (2012). Artemis: An integrated platform for visualization and analysis of high-throughput sequence-based experimental data. Bioinformatics.

[24] Punta M (2012). The Pfam protein families databases. Nucleic Acids Res 40 D290-D301..

[25] Tatusov RL (2003). The COG database: an updated version includes eukaryotes. BMC Bioinformatics.

[26] Schattner P, Brooks AN, Lowe TM (2005). The tRNAscan-SE, snoscan and snoGPS web servers for the detection of tRNAs and snoRNAs. Nucleic Acids Res..

[27] Enright AJ, Van Dongen S, Ouzounis CA (2002). An efficient algorithm for large-scale detection of protein families. Nucleic Acids Res..

[28] Darling, A. C. E., Mau, B., Blattner, F. R. & Perna, N. T. Mauve: Multiple Alignment of Conserved Genomic Sequence With Rearrangements. **14**, 1394–1403 (2004). 10.1101/gr.2289704 PMC442156 15231754

[29] Edgar RC (2004). MUSCLE: Multiple sequence alignment with high accuracy and high throughput. Nucleic Acids Res..

[30] Guindon S (2010). New algorithms and methods to estimate maximum-likelihood phylogenies: Assessing the performance of PhyML 3.0. Syst. Biol..

[31] Bottacini F (2015). Discovery of a conjugative megaplasmid in *Bifidobacterium breve*. Appl. Environ. Microbiol..

[32] Tran TTT, Belahbib H, Bonnefoy V, Talla E (2015). A Comprehensive tRNA Genomic Survey Unravels the Evolutionary History of tRNA Arrays in Prokaryotes. Genome Biol. Evol..

[33] Briner AE (2015). Occurrence and diversity of CRISPR-Cas systems in the genus *bifidobacterium*. PLoS One.

[34] Kunz C, Rudloff S, Baier W, Klein N, Strobel S (2000). Oligosaccharides in H Uman Milk: Structural, Functional, and Metabolic Aspects. Annu. Rev. Nutr..

[35] Sela DA (2008). The genome sequence of *Bifidobacterium longum* subsp. *infantis* reveals adaptations for milk utilization within the infant microbiome. Proc. Natl. Acad. Sci. USA.

[36] Egan M, O’Connell Motherway M, van Sinderen D (2015). A GntR-type transcriptional repressor controls sialic acid utilization in *Bifidobacterium breve* UCC2003. FEMS Microbiol. Lett..

[37] Makino H (2011). Transmission of intestinal *Bifidobacterium longum* subsp. *longum* strains from mother to infant, determined by multilocus sequencing typing and amplified fragment length polymorphism. Appl. Environ. Microbiol..

[38] Conlon MA, Bird AR (2014). The Impact of Diet and Lifestyle on Gut Microbiota and Human Health. Nutrients.

[39] Charbonneau MR (2016). Sialylated Milk Oligosaccharides Promote Microbiota-Dependent Growth in Models of Infant Undernutrition. Cell.

[40] Dirix G (2004). Peptide signal molecules and bacteriocins in Gram-negative bacteria: A genome-wide in silico screening for peptides containing a double-glycine leader sequence and their cognate transporters. Peptides.

[41] Guglielmetti S, Mayo B, Álvarez-Martín P (2013). Mobilome and genetic modification of bifidobacteria. Benef. Microbes.

[42] Ates LS, Houben ENG, Bitter W (2016). Type VII Secretion: A Highly Versatile Secretion System. Microbiol. Spectr..

[43] Das C, Ghosh TS, Mande SS (2016). In silico dissection of Type VII Secretion System components across bacteria: New directions towards functional characterization. J. Biosci..

[44] Cao Z, Casabona MG, Kneuper H, Chalmers JD, Palmer T (2016). The type VII secretion system of *Staphylococcus aureus* secretes a nuclease toxin that targets competitor bacteria. Nat. Microbiol..

[45] Ventura M, Canchaya C, Zhang Z, Fitzgerald GF, Van Sinderen D (2007). Molecular characterization ofhsp20, encoding a small heat shock protein of *Bifidobacterium breve* UCC2003. Appl. Environ. Microbiol..

[46] Ponting CP, Aravind L, Schultz J, Bork P, Koonin EV (1999). Eukaryotic Signalling Domain Homologues in Archaea and Bacteria. Ancient Ancestry and Horizontal Gene Transfer. J. Mol. Biol..

[47] Chylinski K, Le Rhun A, Charpentier E (2013). The tracrRNA and Cas9 families of type II CRISPR-Cas immunity systems. RNA Biol..

[48] Scholz M (2016). Strain-level microbial epidemiology and population genomics from shotgun metagenomics. Nat. Methods.

[49] Asnicar, F. *et al*. Studying Vertical Microbiome Transmission from Mothers to Infants by Strain-Level Metagenomic Profiling. **2**, 1–13 (2017). 10.1128/mSystems.00164-16 PMC5264247 28144631

[50] Makino, H. *et al*. Mother-to-infant transmission of intestinal bifidobacterial strains has an impact on the early development of vaginally delivered infant’s microbiota. *PLoS One* **8**, (2013). 10.1371/journal.pone.0078331 PMC3828338 24244304

[51] Mikami K (2009). Influence of maternal bifidobacteria on the establishment of bifidobacteria colonizing the gut in infants. Pediatr. Res..

[52] Milani C (2015). Exploring vertical transmission of bifidobacteria from mother to child. Appl. Environ. Microbiol..

[53] Lax S (2015). Longitudinal analysis of microbial interaction between humans and the Indoor. Environment..

[54] Barberán A (2015). The ecology of microscopic life in household dust. Proc. R. Soc. B Biol. Sci..

