# Supplementary material for: Genomic diversity and distribution of Bifidobacterium longum subsp. longum across the human lifespan
Source: Sci Rep. 2018 Jan 8;8:85. doi: 10.1038/s41598-017-18391-x (PMC5758520; doi:10.1038/s41598-017-18391-x)
Supplement: Supplementary file 1 — Supplementary Information [file 41598_2017_18391_MOESM1_ESM.pdf]

**-Supplementary Information-**

# Genomic diversity and distribution of *Bifidobacterium longum* subsp. *longum* across the human lifespan

Toshitaka Odamaki[1,2*], Francesca Bottacini[2], Kumiko Kato[1], Eri Mitsuyama[1], Keisuke Yoshida[1], Ayako Horigome[1], Jin-zhong Xiao[1] and Douwe van Sinderen[2]

[1]Next Generation Science Institute, Morinaga Milk Industry Co., Ltd., Zama, Kanagawa, Japan

[2]APC Microbiome Institute and School of Microbiology, National University of Ireland, Western Road, Cork, Ireland

Inventory of Supplementary Information

1. Supplementary Figures and Legends
   Supplementary Figure S1-S5

2. Supplementary Tables
   Supplementary Table S1-S11

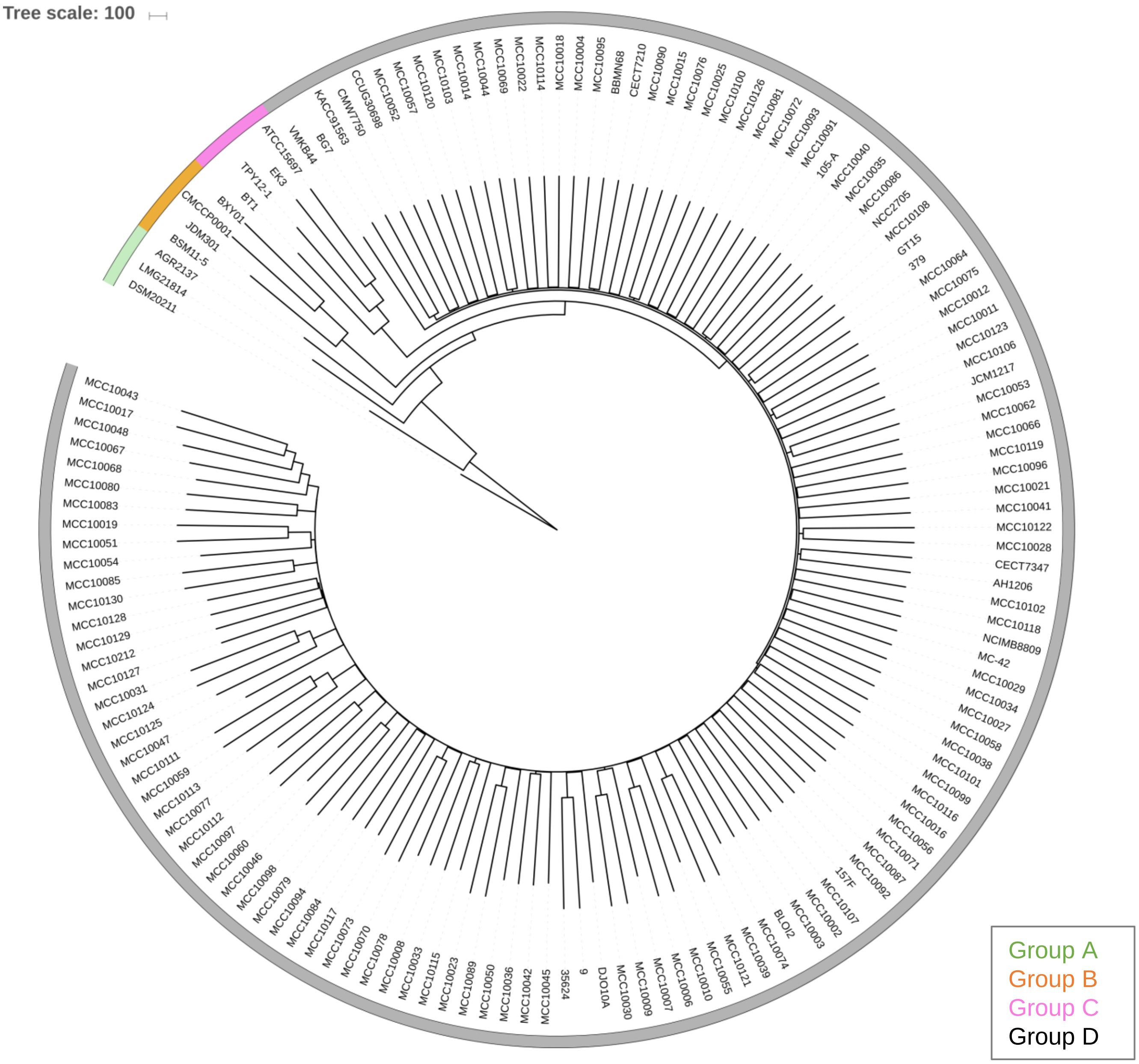


**Supplementary Figure S1**

**Cladgram of *B. longum* strains**

A phylogenetic supertree showing the relationship between 145 *B. longum* strains analysed in this study. The tree was calculated based on the nucleotide sequence of 642 single-core gene families among all strains analysed in this study. Group A and B are composed of *B. longum* subsp. *suis* strains. Strains in Group C are *B. longum* subsp. *infantis*. All of strains isolated in this study (MCC strains) and *B. longum* subsp. *longum* are clustered together in Group D.

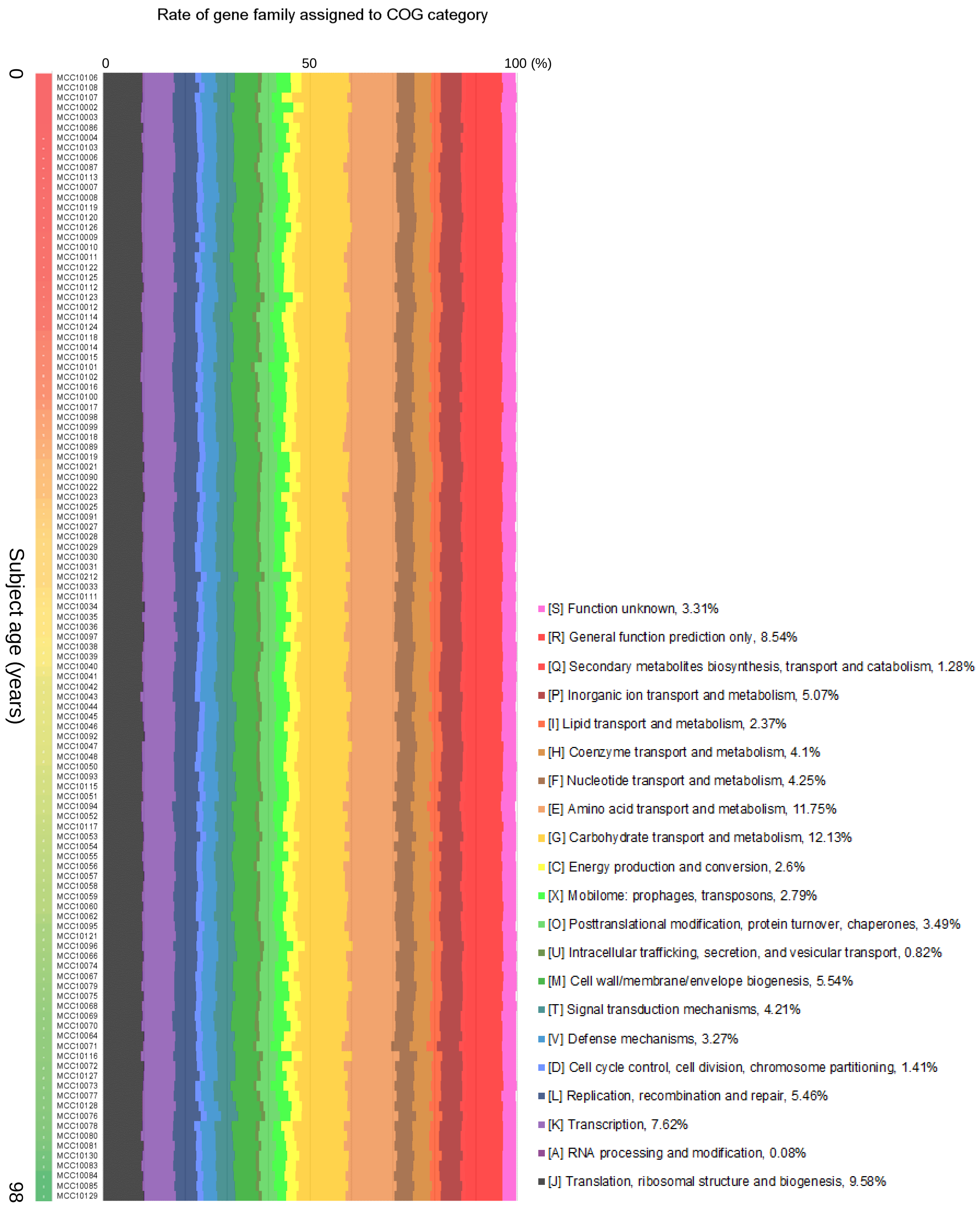


**Supplementary Figure S2**

**Cluster of Orthologue (COG) classification of 113 newly isolated *B. longum* subsp. *longum* strains**

Heatmap showing the COG classification of *B. longum* subsp. *longum* computed ORFs. Strains are sorted by subject age. For each COG entry, the average percentage of hits among *B. longum* subsp. *longum* has been indicated in the legend.

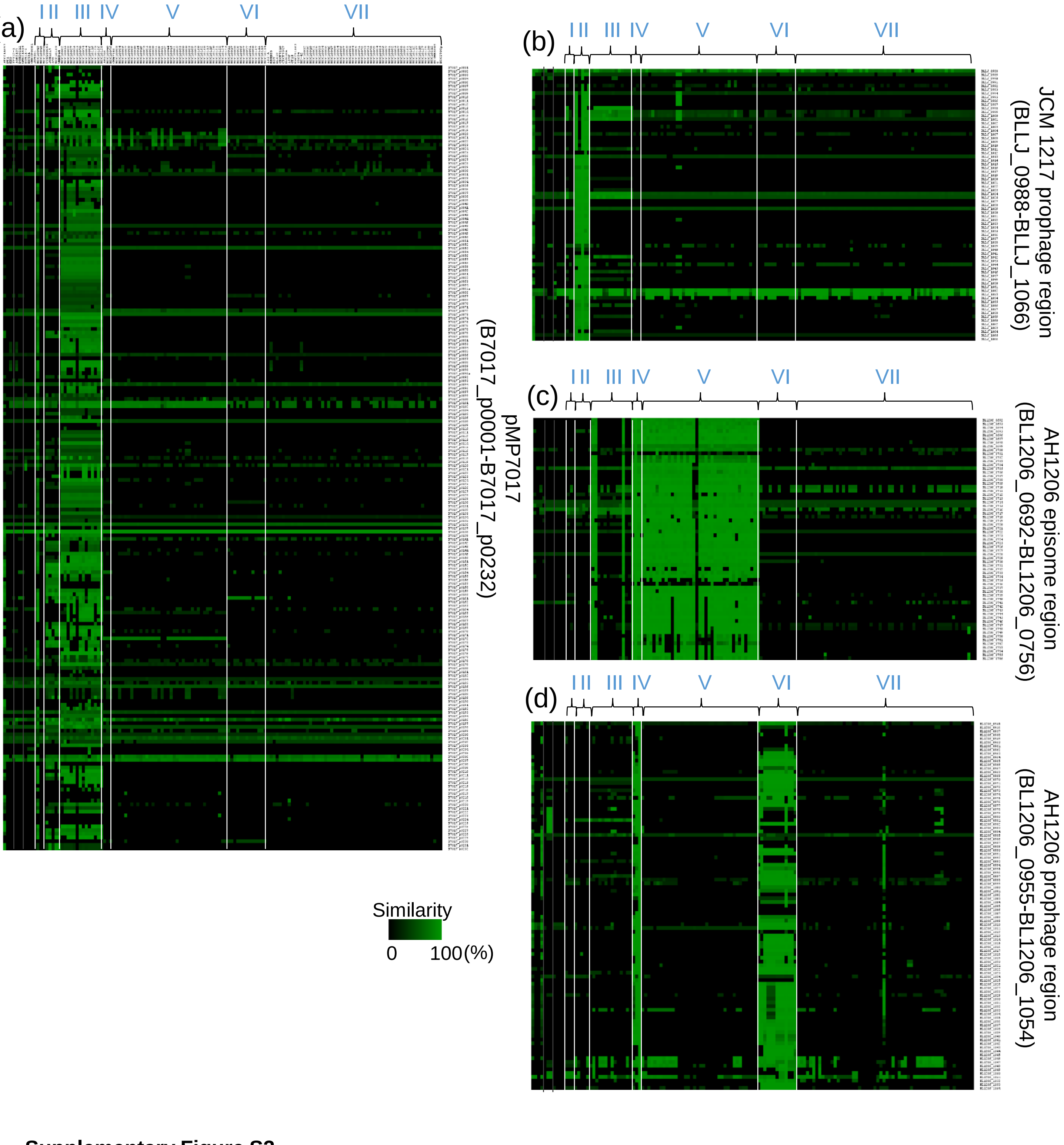


**Supplementary Figure S3**

**Matrix of blastn result against prophage, integrated episome or megaplasmid sequences**

Heat map represents the similarity percentage of blastn result of genes belonging to the *B. longum* mobilome. Blue Greece indicates the longum cluster in Figure 2. (a) Megaplasmid pMP7017 homologues detected in 1 and 14 strains belong to longum cluster I and III, respectively. (b) Homologues of *B. longum* subsp. *longum* JCM1217 prophage region (BLLJ_0988-BLLJ_1066) found from 5 strains in longum cluster II. (c) Homologues of *B. longum* subsp. *longum* AH1206 episome region (BL1206_0692-BL1206_0756) detected in 4, 3 and 37 strains belong longum cluster III, IV and V. (d) Homologues of *B. longum* subsp. *longum* AH1206 prophage region (BL1206_0955-BL1206_1054) detected in all strains belonging to longum cluster IV and VI.

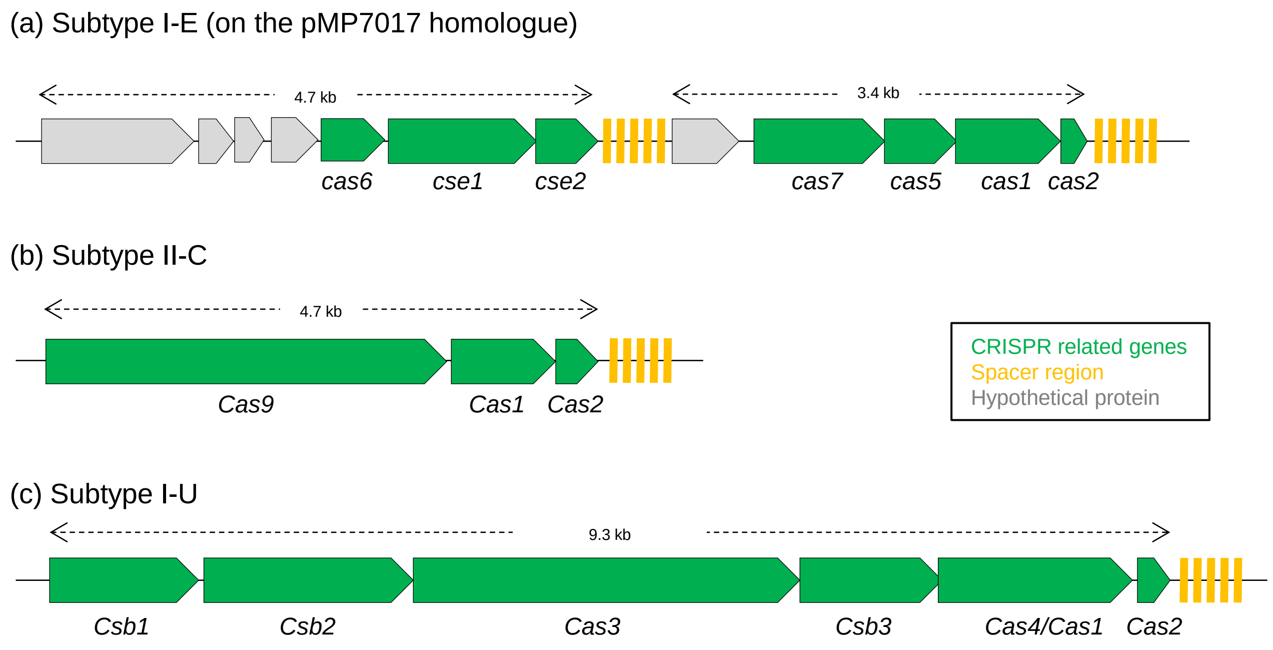


**Supplementary Figure S4**

Subtype of CRISPR-cas system found in this study.

(a) Subtype I-E were located on the predicted megaplasmid sequences in 14 strains. (b) Subtype II-C without tracrRNA were located on the chromosome sequences in 39 strains. (c ) Subtype I-U was located on the chromosome sequences of MCC10117 strain. Each strain was shown in Supplementary Table S7.

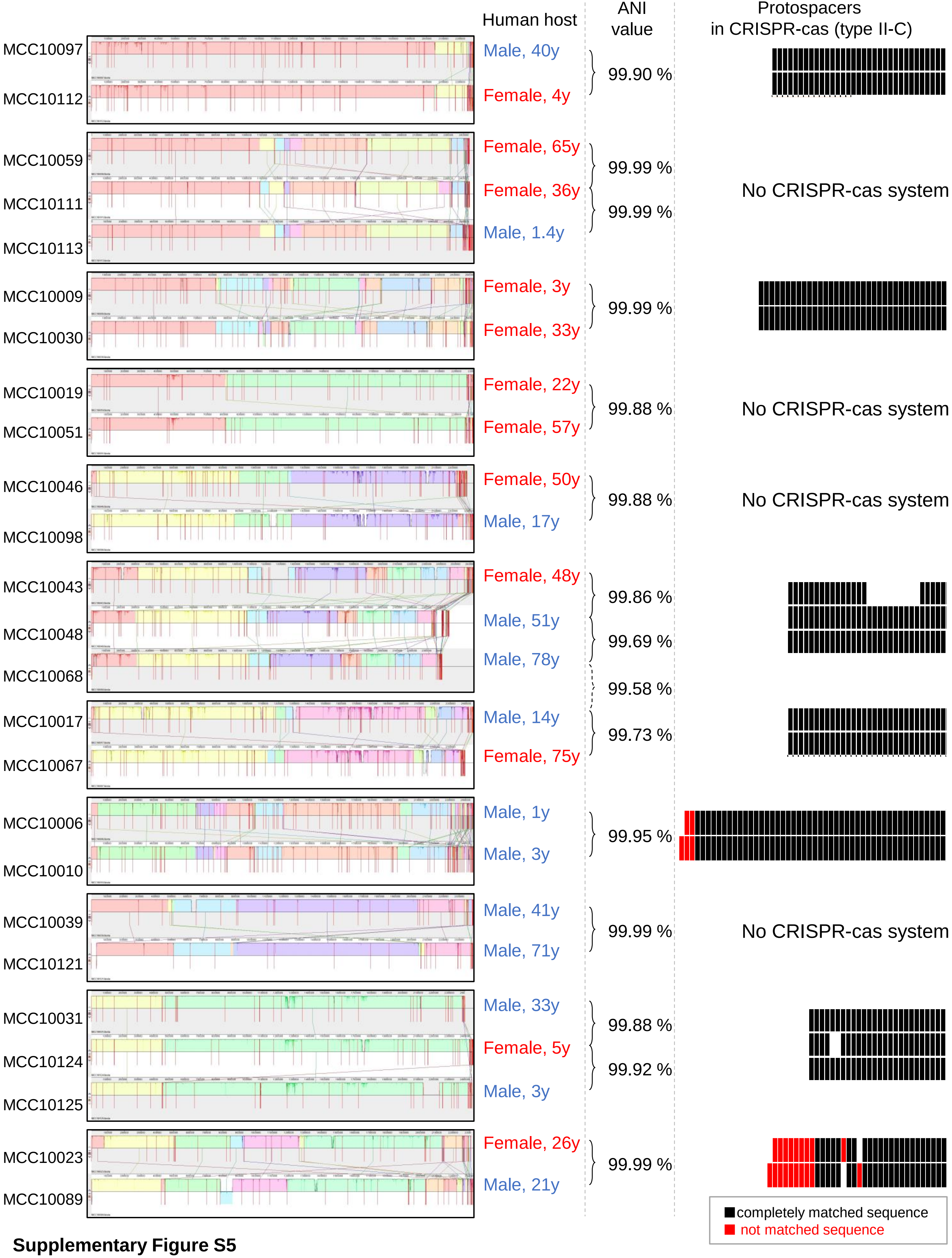


**Supplementary Figure S5**

**Sequence similarity between strains predicted to be transmitted among family members**

Mauve whole nucleotidic alignment performed to assess the similarity between transmitted strains. Coloured boxes represent Mauve-deduced linear collinear blocks (LCB), while white gaps represent insertions and deletions. Vertical blocks represent protospacers in CRISPR-cas system, where the black and red colours indicate completely matched or not matched sequence between transmitted strains, respectively.
